# Profile: Vanessa Cameron – 36 years at the Royal College of Psychiatrists

**DOI:** 10.1192/pb.bp.116.055459

**Published:** 2016-12

**Authors:** Rob Poole, Catherine A. Robinson

## Abstract

On 16 December 2016, Vanessa Cameron retires as Chief Executive of the Royal College of Psychiatrists. She started working there in September 1980 and in 1984 she became Secretary of the College, the role that preceded chief executive. The College was formed in 1971, so Vanessa has been present for most of its lifetime. It has been a period of continuous change that has seen psychiatry leave the old mental hospitals, expand considerably in the late 1990s and early part of the 21st century, and come under huge pressure more recently. Although she has never worked within mental health services, Vanessa has been at the heart of British psychiatry for 36 years. She was awarded an MBE in the 2013 New Year's Honours list for services to psychiatry.

We interviewed Vanessa at 21 Prescot Street on 3 August 2016.

**Figure:**
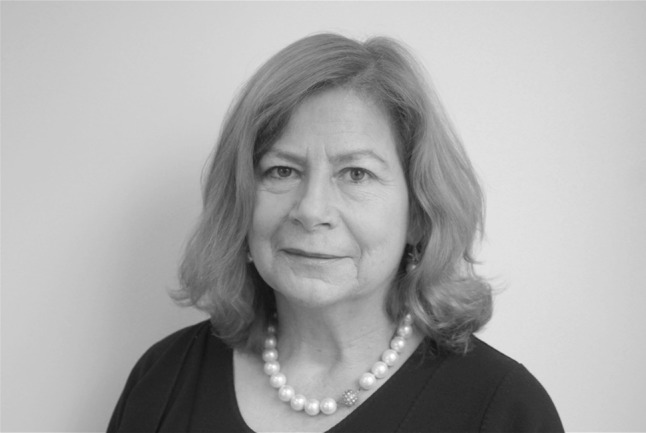
Vanessa Cameron, Secretary of Royal College of Psychiatrists and Chief Executive, 1984-2016

## Early life

Vanessa was born in Norfolk, Virginia, USA, where her father was posted with the Royal Navy. It seems that she was a quintessentially English baby; her father vetoed an early offer of a modelling role in a Pears Soap advertisement. The family returned to the UK when she was 2 years old.

Her father was the son of the organist and choirmaster of Lichfield Cathedral. Vanessa describes him as a ‘clever man who grew up in a Trollopian environment’. He was supported by the Royal Navy to attend Trinity College, Cambridge. He became a mechanical engineer, and later worked in military intelligence. Her mother was a rather flamboyant Highland Scot. She was a former Communist and an early Scottish nationalist. Vanessa has an elder sister, Fiona, who married a submariner, and a younger brother, Charles, who owns a hotel in the West Country. Vanessa remains very close to her siblings and their offspring.

Like most naval families, they moved frequently, although ‘not to very exotic places, mainly naval ports along the South Coast’. She was sent to boarding school to provide stability. She did very well academically until she was studying for her A levels. Although it was not recognised at the time, her performance suffered because she was experiencing early symptoms of bipolar affective disorder. Her academic workload was reduced and ‘it was generally agreed that I wouldn't be able to cope with university, something that I have been compensating for ever since’.

She wanted to get away from the naval environment where she had grown up, and at 18 she left home to go to work in Oxford (‘I always wanted to be close to really clever people’). She worked in Blackwell's bookshop, ‘earning the princely sum of eight pounds per week. I spent a lot of time looking for shoplifters to earn the reward of five pounds per shoplifter’. After this, she went to work at St Edmund Hall as the assistant college secretary, dealing with admissions and all kinds of student matters.

## From setback to the College

In 1972, Vanessa took up a post at the United Nations in Geneva, working for two Soviet diplomats. The Cold War was in full swing and ‘to put it mildly, it was interesting’. They were working on the first Middle East Peace Conference, and her role was to ensure that everything that left the office was written in good English. Vanessa became involved in a difficult security situation and she became very unwell. She was hospitalised and given the diagnosis of ‘manic depression’. She says that having a diagnosis and an understanding of what had been wrong was reassuring. She left her job on health grounds and returned to the UK.

She describes this as ‘a very black period. I had no job, no income, no home and I was living with my parents, who were about to divorce’. Fortunately, things gradually improved. She secured a post at University College Oxford as the assistant secretary, ‘dealing with admissions, degree ceremonies and all kind of things’. A couple of years later she was promoted to assistant domestic bursar with responsibility for developing conference income during the vacations.

During this period she met her husband, Jonathan, who is now a media lawyer and Emmy-nominated film producer. He was studying law at Brasenose College. It was his second degree; he had read English and Philosophy at Edinburgh University. When he left Oxford to do his articles, he and Vanessa relocated to London. Searching for a job, Vanessa eventually arrived, more or less by chance, at 17 Belgrave Square for an interview with the Registrar, Professor Gerald Timbury, and the Secretary Natalie Cobbing. This was not a choice that was determined by her own experience of illness: ‘I wasn't thrilled that it was the Royal College of Psychiatrists. Although I had had a good experience of psychiatry, I really wanted to put my experience of mental illness behind me. I was, however, thrilled to have my own secretary, Sue Duncan. Sue, who still works with me, has been my constant colleague; more like my kid sister than my secretary. She's been loyal, utterly discreet, and a source of practical advice. She's a great friend, and I'll miss her hugely when I retire.’

Vanessa came to the College with a positive view about psychiatry's ability to help people: ‘My life was turned around by my psychiatrist, Maurice Lipsedge. He was so wise and so clever, mixing this with such compassion. He became my model psychiatrist. It would be great to thank him publicly, if that is allowed’.

Vanessa and Jonathan had a flat in Notting Hill. Jonathan is Jewish and Vanessa converted to Judaism. This was a serious commitment, involving years of classes and synagogue attendance. Neither of them is particularly religious, but they wanted their children to grow up in one faith. Vanessa was drawn to Judaism and loved its family traditions. The couple tried to have children, which meant that Vanessa had to discontinue lithium. Each time she did this, she would become ill and needed hospitalisation. ‘Eventually, we both agreed that the attempt to have children was ruining our lives and careers and risked destroying our marriage. We have never looked back.’

When she arrived in 1980, the College had just 28 staff. Vanessa's job was demanding. She looked after all of the faculties, the Public Policy Committee, the House Committee and the working party on the White Paper on the Mental Health Act. Vanessa describes the College at that time as being ‘rather stuffy’, a predominantly male environment, ‘like a gentlemen's club’. Pipes were smoked in Council, which closed with alcoholic drinks.

Staff at all levels had administrative rather than managerial roles. Their relationship with members was somewhat remote. Vanessa did not especially enjoy the environment she was working in and she decided to leave. She was serving her notice when Natalie Cobbing died suddenly in January 1984. Professor Ken Rawnsley was President at the time, and he asked Vanessa to act up as Secretary until a permanent appointment was made. Vanessa was just 32 years old at the time, ‘too young for the job, really’. She enjoyed acting up and began to see that changes could be made, so she applied for the permanent post and was duly appointed in June 1984.

## The changing nature of the College

The College has grown enormously during Vanessa's tenure, culminating in 2013 in the move from the cramped grandeur of 17 Belgrave Square to more modern, and much larger, premises at 21 Prescot Street. It now employs over 200 staff. Vanessa feels that the College has changed from a generally rather conservative organisation to a much more relaxed establishment with a generally liberal, or even left-wing, atmosphere. Business is conducted in a polite and civilised way. She believes that, in the past, some other medical Royal Colleges have tended to conduct business in a rather more robust fashion. Unlike them, the College has never generated rumours of fisticuffs at meetings.

For all of its early medical conservatism, the College never went in for the traditional trappings of Royal Colleges, such as extensive wine cellars, ceremonies and robes for members. According to Vanessa, this was largely due to the influence of the Association of Psychiatrists in Training, led by young radicals such as John Gunn, Anthony Clare and John Hamilton. They negotiated with Sir Martin Roth (the first College President) for a more down-to-earth organisation, with trainee representation on almost every committee. Consequently, trainees have been strongly influential ever since. Many members of the Trainees' Committee have remained involved with the College throughout their careers, and quite a few have gone on to take leadership roles.

When robes and ceremonies were finally introduced, it was in response to the wishes of a new generation of trainees who were more attached to traditional rites of passage than their predecessors. In Vanessa's view, the College's support for the junior doctors during their recent contract dispute with NHS England and the Secretary of State for Health, Jeremy Hunt, demonstrates the continuing strength of the relationship between trainees and the College.

Vanessa feels that a key change in the College was due to the influence of Sir Roy Griffiths, at that time Deputy Chairman of (retailer) Sainsbury's and adviser to the Thatcher government, who recommended the introduction of general management into the NHS:

‘There was a report, called the Griffiths Report, which people were horrified by, […] [complaining] it was written by “a grocer”, or at least somebody who worked for Sainsbury's. It had a huge impact on the College. It recommended massive changes to NHS structures, but also that doctors should become managers. For many years after there were anecdotal stories of money being wasted on management but it all calmed down in the end. I think that it has been very beneficial to psychiatrists to be involved in medical management. If you look at the discussions that we have now compared with the discussions that we had in the early 1980s, there is a tremendous change. I think that managerial proficiency was beneficial in the long run. When I look at the minutes of Council meetings now, there are many references to what the directors or the chief executive has done, but back in the 80s, when it was very much a “gentlemen's club”, it was a very different relationship. College staff were seen as clerical assistants or whatever. As psychiatrists were introduced to management at work, they came to respect the management that they saw in the College.'

## Presidents

Since its formation in 1971, the College has had 15 presidents. Vanessa has known them all, and her tenure has coincided with the term of office of 13 (the exceptions being Professor Sir Martin Roth and Professor William Linford Rees). Vanessa thinks that the College is truly representative of psychiatry, with high levels of engagement of the membership with its work. Consequently, much of the important business of the College carries on irrespective of the qualities of the president. The president is not necessarily the public face of psychiatry. Nonetheless, each president brings something special, and Vanessa regards some as having been particularly successful.

She holds the late Professor Ken Rawnsley in especially high regard. He was responsible for a number of key developments in the first 15 years of the College's existence. He was the first Dean of the College, and President from 1981 to 1984. ‘He set up what everyone thought was the jewel in the crown, what was called the Central Approval Exercise. This involved teams of two senior doctors and a trainee visiting hospitals around the country, approving training. It was the first systematic approval process. What Ken Rawnsley set up […] probably did more to improve the practice of psychiatry than anything else. It came to a very sudden end in the late 1990s when Alan Milburn was Health Secretary. The Royal College of Surgeons closed down a surgical unit in his constituency and he shut down the whole approval system. It seemed like an act of revenge. It was really sad for psychiatry, because we would never have closed down a unit, we would go back and help them develop. Our system was the best across medicine but it went with the rest. Nonetheless, that was Ken's first major achievement. He was very concerned about the political abuse in psychiatry, particularly in the Soviet Union. He was instrumental in passing a resolution at the College that the USSR should be expelled from the World Psychiatric Association. In the end the Soviet Union withdrew before they could be expelled, which was one of their tactics. I think that it was a fine thing that the Royal College did in the early days, really fought against political abuse, not just in the USSR but also in China, Japan, South Africa, Romania, all those countries. This was all started by Ken. He ran […] the National Counselling Service for Sick Doctors, a confidential service across medicine linked to the GMC. It looked after doctors who were unwell, a little bit like the College's Psychiatrists’ Support Service now, but this was across medicine, not just for psychiatry. I would keep a whole list of psychiatrists who would be able to help and I would get phone calls from people asking for psychiatrists to help doctors.

‘[Ken] also produced the first report on psychiatric training and practice in a multi-ethnic society and his document lasted for a very long time, although there were later two more versions of it. There were huge discussions among the working groups. People said that the Irish should be referred to as Black because anyone who was a minority was Black. There were huge arguments, as you can imagine.

‘I would say one more thing about Ken. When I was very unwell and my husband was struggling to look after me, Ken took me to his house. He and his wife looked after me and gave my husband a break for a few weeks. He was a truly compassionate and good man.'

Despite her obvious admiration for Professor Rawnsley, Vanessa is not nostalgically over-attached to the distant past: ‘Maybe it is because I am now a similar age to, or even older than, the last three presidents, Dinesh [Bhugra], Sue [Bailey] and Simon [Wessely] … not only have I been hugely impressed by their achievements as presidents and beyond, but I count them as friends as much as colleagues. The past 9 years have been particularly happy for me at the College.’

## Mental health acts

There have been two major revisions of English mental health legislation during Vanessa's tenure, together with the introduction of the Mental Capacity Act and the Deprivation of Liberty Safeguards. Vanessa feels that the College was quite successful in influencing the Mental Health Act 1983, and its subsequent amendments, mainly through persistent hard work by Professor Robert Bluglass in the first instance, and more recently by Dr Tony Zigmond.

Larry Gostin, an American civil liberties lawyer, was Legal Director of MIND (UK mental health charity) during the drafting of the Mental Health Act 1983. In contrast to good relationships within the broad mental health community in the 21st century, the relationship between Gostin and the College was extremely strained, if not actually antagonistic. On reflection, Vanessa thinks that Gostin probably had a positive effect on UK mental health law. She used to meet him at the All-Party Parliamentary Group on Mental Health: ‘I remember Larry Gostin at his last meeting. His mother came over from America, she was so proud of him. I saw a different side of him. He was actually quite a nice guy. Maybe things needed to change and he helped change them.’

## Campaigns and external relationships

Vanessa recalls that the College was extremely reluctant to engage with the press and other media in the early years. One of the first issues she raised at Council as Secretary was the need to become less defensive in order to get a message across to the public.

The first sign of a change was the publication of a book, *Alcohol, Our Favourite Drug*: ‘The book introduced the whole concept of using units of alcohol. Units hadn't been discussed publicly before. It was a public relations dream. The book had about ten messages about drinking, drinking when you are pregnant, what units were – amazing. We got a PR company to help launch [it]. It was so successful that the company had to sack us as a client, because a major brewer said, “if you go on working with the Royal College of Psychiatrists, you do not work for us”. We were sacked, but we were off on a journey with our press and media work. Now we use the press and media whenever we can. That is a major change. We went on to launch four public education campaigns. The first was the Defeat Depression campaign. It was aimed at GPs and informing the public that depression was treatable. It lasted 4 years. Robin Priest [Registrar 1983-1988] led it.’

The Defeat Depression campaign proved controversial: ‘There was a general view expressed for years after that we had been taken for a ride by the pharmaceutical companies. I don't think we were particularly. There were some great hearts in that campaign. I think it was successful and I don't think Pharma made any difference really. It was a good campaign, well planned.’

These days, the relationship between the College and Pharma is distant: ‘I think that these days we have an appropriately puritanical relationship with Pharma. After the Defeat Depression campaign we produced guidance that said we wouldn't use Pharma for any public education activities. Psychiatrists can give medication to people against their will and we cannot be in the pockets of the Pharma companies.’

It was not always thus: ‘In the 80s, I remember going to my first American Psychiatric Association meeting. The President and I got into the economy class and all the other psychiatrists went into business class. That wouldn't happen now. The whole relationship with Pharma has changed and I think it is right.

‘We used to have some [Pharma-sponsored] lectures in the 80s, as some professors were getting money from them for their research. If the country had proper research funding, you wouldn't need Pharma funding and you wouldn't have professors of psychiatry having to get money from Pharma. So you know, that makes me quite angry that there isn't sufficient funding and we have to go elsewhere to [find it]. But the College has done its best to define its own rules and regulations over Pharma and we get less than 1% of our income from Pharma, if that, at the moment.’

Professor Arthur Crisp led the next public education programme. It was an anti-stigma campaign called Every Family in the Land: ‘It was very scientific, as you would expect from Arthur Crisp, with attitude surveys at the beginning and at the end. We got a major advertising company to pay for the short film we made and Warner Brothers agreed to show that film in their cinemas for 6 weeks. You watched it when you were eating your popcorn waiting for the main film to come on. It was saying that one in four people will have mental illness at some stage in their life.’

The third campaign, in 2004 to 2005, was called Partners in Care and was led by Mike Shooter (President 2002–2005). It was conducted jointly with the Carers Trust. Baroness Sheila Hollins (President 2005–2008) led the fourth campaign. It was called Fair Deal and was concerned with parity of esteem.

In parallel with this, the College has become much more sophisticated in its efforts to influence government. In the early years, ‘I don't think the College was as well organised as it is now. It was still sort of amateur. It did very well in some things, and produced very considered responses to government papers. It wasn't until we had resolved our relationship with the press, and then when we had a policy unit, that we had a much more structured and effective way of influencing government. Before that, we mainly had an impact on mental health legislation. When Thatcher was in power, we couldn't stop them. Thatcher's government wanted to change things within the health service. Then Tony Blair came in, and his government wanted to change things even more and I don't think anybody could have influenced them, apart from the few psychiatrists who were advising the government. I think we were just not as savvy as we are now.’

The pace of change under New Labour could be exhausting: ‘Changes came so fast upon each other, people would say “oh no, another consultation paper”, and we all got consultation fatigue. There seemed to be a plethora of policy papers. We seemed to start having an influence again when the Mental Health Act was being looked at by Genevra Richardson (then Professor of Public Law and chair of the expert group that reviewed mental health legislation in the late 1990s). Apart from that, change was being driven from outside of the College and the profession, and we constantly had to respond. We were on the ‘back foot’ and that is probably why Sheila Hollins said in 2008 we must have a policy department. That was a very good decision.’

When community treatment orders were first proposed, the College opposed their introduction. This led the College into a formal campaigning group alongside service users' organisations for the first time.

‘We had a Patients and Carers Committee from about 2001. Its formation was a reflection of what was going on externally, as these things often are. I remember a chief executive of another [medical] Royal College saying “oh well, if the Psychiatrists can involve their patients and carers then it should be easy for the rest of us”. A bit insulting. I adopted my zero tolerance at that stage, so people stopped saying that kind of thing after a while.

‘The relationship with users and carers really changed during Mike Shooter's time as president. He was very open about having a mental illness and it made a huge impact. I think things developed from there. He really embraced working with patients and carers and from then onwards I think the College has had a very powerful relationship [with them]. We joined the Mental Health Alliance, which consisted of all the major charities, quite in contrast to what happened with the 1983 Act. It became apparent that psychiatry was very, very powerful when it allied itself with patient groups and carers. Mike Shooter would say that the partnership was unstoppable. He wanted to go a step further and change to a College of Mental Health. He wanted nurses and social workers, psychologists and psychiatrists to all join as equals in a new, reformed College, but that got almost no support. I think he is still very disappointed to this day. Psychiatrists felt that they needed an organisation that was just for them.

‘Despite that, now we have a situation where almost every committee in the College, and all our quality improvement visits, include a service user and carer. It certainly is a complete reversal.’

## The future

Vanessa feels that she leaves the College at a time when it is more professional and business-like than ever before. She sees a pattern of increasing sophistication in the relationship with the membership, the government and the general public. For the first time, mental health is on the agenda of all of the major political parties, and public debate about it is not dominated by misleading and sensational headlines. The Annual Meeting has grown into the highly successful International Congress, which is addressed by government ministers and the chief executive of NHS England. There are many serious challenges ahead, but the College is well prepared.

At this late stage, Vanessa is prepared to disclose that she is a lifelong social democrat and that she has republican sympathies, which is perhaps ironic in someone who has led a Royal College for so long. She has been proud to work for the College. The organisation's close match with her own values has been a major factor in her loyalty. As for her retirement plans:

‘During 2017, I will be doing some work in Geneva with the World Psychiatric Association. I shall continue my work for the Ministry of Justice as a Specialist Lay Member of the Mental Health Tribunals, and I have been invited to join the Board of the Global Initiative on Psychiatry, which deals with human rights in mental health. I have one or two other irons in the fire. I shall not be bored!’

